# Effects of ambient temperature during the nestling stage on a stress indicator in nestling pied flycatchers *Ficedula hypoleuca*

**DOI:** 10.1007/s00484-021-02199-6

**Published:** 2021-10-07

**Authors:** Joanna Skwarska, Agnieszka Podstawczyńska, Mirosława Bańbura, Michał Glądalski, Adam Kaliński, Marcin Markowski, Jarosław Wawrzyniak, Piotr Zieliński, Jerzy Bańbura

**Affiliations:** 1 Department of Experimental Zoology and Evolutionary Biology, Faculty of Biology and Environmental Protection, University of Łódź, Banacha 12/16, 90-237 Łódź, Poland; 2 Department of Meteorology and Climatology, Faculty of Geographical Sciences, University of Łódź, Narutowicza 88, 90-139 Łódź, Poland; 3 Museum of Natural History, Faculty of Biology and Environmental Protection, University of Łódź, Kilińskiego 101, 90-011 Łódź, Poland; 4 Department of Ecology and Vertebrate Zoology, Faculty of Biology and Environmental Protection, University of Łódź, Banacha 12/16, 90-237 Łódź, Poland

**Keywords:** *Ficedula hypoleuca*, Nestlings, Stress indicator, Temperature indicators, Precipitation

## Abstract

Long-term and short-term changes in ambient temperature can cause stress in birds, leading to changes in the level of hematological parameters. The H:L ratio (heterophil-to-lymphocyte ratio) is a hematological index that allows for the assessment of the stress induced by environmental changes, including weather conditions. In this paper, we examined the influence of temperatures and the sum of precipitation on the health of nestling pied flycatchers (*Ficedula hypoleuca*) by using the H:L ratio reflecting the body’s response to stress. All examined temperature indicators influenced the H:L ratio, yet the average value of daily minimum temperature during the first 12 days of nestling life had the strongest influence, maximum temperature had the weakest effect, while precipitation had no significant influence. Our research indicates that even a small increase in temperature caused a stress reaction in nestling pied flycatchers, which was reflected by an increase in the H:L ratio. The increase in the stress index (H:L ratio) was probably a result of poor weather conditions (precipitation, low temperature), which prevented the adult birds from actively foraging and properly feeding the nestlings.

## Introduction

Birds are particularly vulnerable to changes in ambient temperatures due to their small body size (McKechnie and Wolf 2010). Even a slight increase in ambient temperature can negatively affect the fitness and survival of nestlings (Shilov 1973; O’Connor 1984; Saito and Grossmann 1998; Thomas et al. 2001). Nestlings can protect themselves from increased ambient temperatures losing heat by means of convection rather than by evaporation. Homeotherms can drive a decrease in resting metabolic rate (MR) to reduce the production of metabolic heat and also to reduce the need for water used in thermoregulatory processes as soon as the ambient temperature approaches body temperature and finally to balance it (Thomas et al. 2001). Furthermore, adult birds can accelerate heat exchange between their bodies and the environment through air sacs (El-Tarabany 2016). Air sacs promote the circulation of air on the lung surface, contributing to increased gas exchange and consequently leading to heat loss through evaporation (Fedde 1998). Studies on poultry showed that hyperthermia (overheating of the body) contributes to lower food intake, and this consequently leads to lower growth rates (Rodríguez and Barba 2016). Low food intake increases the body energy requirements for physiological processes, and this leads to weight loss (Routman et al. 2003; Catry et al. 2015). However, studies conducted on nestling great tits (*Parus major*) show that days with average temperatures above 12 °C (classified as hot) also contributed to increased parental costs of feeding nestlings. This may have been due to the increased food requirements of young birds to compensate for thermoregulatory costs (Royama 1966; Barba et al. 2009).

Precipitation can also negatively affect nestling provisioning rates and nestling success (post-fledging survival) (Facey et al. 2020). Particularly for insectivorous birds, periods of rain and low temperatures during the breeding season result in poorer nestling growth and higher mortality of older nestlings (Järvinen and Ylimaunu 1986; Zając 1995; Catitti 2018). This is confirmed by observations on, e.g., nestling pied flycatchers (*Ficedula hypoleuca*) (Siikamäki 1996) and Lapland longspurs (*Calcarius lapponicus*), in which precipitation negatively affected growth and body mass (Pérez et al. 2016).

This stress, lasting for days (ca. 4–5 days) or weeks, can affect the activity of the immune system and increase susceptibility to disease, leading to changes in the level of hematological parameters (Suorsa et al. 2003; Kaliński 2014, 2015; Glądalski et al. 2015). One of the possible responses to long-term stress in vertebrates is a change in the numbers and the relative frequencies of leukocytes in the blood (Ots and Hõrak 1996). Stress factors cause the heterophil concentrations to increase, while lymphocyte numbers decrease in the blood (Ellis et al. 2012), and as a result of acute stress reaction, increased stress index, the H:L ratio, is observed (Vleck et al. 2000). Studies conducted by El-Tarabany (2016) confirm that heat stress contributes to a decrease in leukocyte and lymphocyte counts. The occurrence of lymphopenia is the body’s response to the release of glucocorticoids, which dissolve lymphocytes in lymphoid tissues (Gross and Siegel 1983). Studies by Nathan et al. (1976) and Mashaly et al. (2004) also confirm a decrease in leukocyte counts and antibody production in birds exposed to high temperatures (42 and 35 °C, respectively). In turn, high temperature causes an increase in the number of heterophils. This is related to the release of adrenocorticotropic hormone, which contributes to the increase in heterophil synthesis in the bone marrow (Al-Murrani et al. 1997). Thus, high temperatures cause a significant increase in the H/L ratio. The H:L ratio is a hematological index that allows for the assessment of the body response to stress induced by weather and/or other environmental changes, e.g., fragmentation of the breeding area (Suorsa et al. 2004; Krams et al. 2010). This parameter allows us to easily estimate the efficiency of the immune system as well as the individual health status of nestlings and adults in wild bird populations (Davis et al. 2008; Skwarska 2018).

The aim of this study was to examine the influence of weather conditions (temperatures and a sum of precipitation) on the health status of pied flycatcher nestlings. The pied flycatcher is a small, insectivorous hole-nesting passerine (Lundberg and Alatalo 1992). It is a single-brooded species, which migrates from its wintering grounds in sub-Saharan Africa to the breeding grounds in the Western Palearctic. Pied flycatchers are one of the best studied passerine birds that are used as model species in animal ecology studies. Pied flycatchers breed in cavities as well as in nest-boxes (Moreno et al. 2005), which allows relatively easy access to the nest to collect samples for analyses from nestlings as well as adult birds (Bell et al. 2017). Our main prediction is that an increase in ambient temperature may potentially exert a negative effect on the health and condition of nestlings. We expect the H:L ratio to elevate as the ambient temperature increases. We also suppose that an increase in precipitation will contribute to this increase in the condition index. Therefore, we treat the increase in the level of H:L ratio as a reflection of the body’s reaction to stress due to the increase in ambient temperature in combination with precipitation.

## Methods

This study was conducted in 2015–2019 as part of a long-term project concerning secondary cavity nesters in central Poland. The study plot (c. 145 ha) was located in the central part of the Łagiewniki Forest (51° 50′ N; 19° 29′ E), bordering the NE suburbia of Łódź. The main study area is located in a mature mixed deciduous forest, with oaks (*Quercus petraea*) and (*Quercus robur*) as the dominant tree species (Marciniak et al. 2007), with small-leaved limes (*Tilia cordata*), sycamore maples (*Acer pseudoplatanus*), Norway maples (*Acer platanoides*), common hornbeam (*Carpinus betulus*), European beech *(Fagus sylvatica*) as subdominant species, and with a minor admixture of Scots pine (*Pinus sylvestris*), European larch (*Larix decidua*), Eurasian aspen (*Populus termula*), and silver birch (*Betula pendula*) (Kurowski 2001).

The study area was supplied with 300 wooden nest-boxes, which were placed at a distance of at least 40 m between each other. From early spring, the nest-boxes were visited once a week to identify the bird species and during which all breeding attempts were recorded. All field procedures were carried out between 9.00 AM and 2.00 PM. In the first place, nest-boxes were occupied by great tits (*Parus major*) and blue tits (*Cyanistes caeruleus*), while pied flycatchers, being long-distance migrants, occupied those boxes which remained empty after their arrival to the breeding site (Skwarska et al. 2012).

The nestling period is a very demanding stage in the life of birds, especially in altricial birds whose endothermy develops after several days of nestling life (Shilov 1973; Brightsmith 2005; O’Connor 1984). In the case of nestling pied flycatchers, the first 12 days after hatching are known to be a temperature-sensitive period (Shilov 1973). Studies on nestling great tits show that 3–4-day-old individuals start to regulate their body temperature, but homeothermia stabilizes only after 10 days of life (Mertens 1977). For these reasons, we calculated weather indicators that covered 12 days starting with the date of hatching in individual broods of pied flycatchers, which we used to test for an influence on the H:L ratio of nestlings. The weather indicators were as follows: mean daily temperature for 12 days (T_mean_12_), mean maximum daily temperature for 12 days (T_max_12_), mean minimum daily temperature for 12 days (T_min_12_), and total precipitation for 12 days (P_12_). Data on daily mean temperature, daily maximum temperature, daily minimum temperature, and precipitation were collected at Dobra-Nowiny (51° 51′ 20″ N, 19° 33′ 34″ E, altitude 195.5 m a.s.l, using automatic weather station of Department of Meteorology and Climatology, University od Łódź), in a location 5 km from the study site.

Data on 185 pied flycatcher nestlings, from 65 broods (2015, 7 clutches, 21 nestlings; 2016, 12 clutches, 35 nestlings, 2017, 13 clutches, 37 nestlings; 2018, 11 clutches, 34 nestlings; 2019, 22 clutches, 58 nestlings), were used for the analyses.

On day 12–14, in isolated cases 10–16 after hatching, the nestlings were banded with individually numbered rings and measured (the length of the wing to nearest 1 mm and were weighed to the nearest 0.1 g). Three nestlings were blind-drawn out of the same-age individuals from each brood to collect blood samples from the ulnar vein directly to make blood smears to count frequencies of leukocytes (Bańbura et al. 2013). Blood smears were used to determine the H:L ratio, with each smear being air-dried, fixed with methanol (100%), and stained in the laboratory with Microscopy Hemacolor kit (Merck Chemicals) (Bańbura et al. 2013; Skwarska et al. 2019). The next step was to count individual fractions of white blood cells within 100 cells under oil immersion using the microscope at × 1000 magnification. All counts of leucocyte fractions were made by the same person (JS), with independent counts of H:L ratio in the same smear being highly repeatable (r_i_ = 0.82; F_1,10_ = 3.63, *p* = 0.022).

The H:L ratio was log-transformed (log(1 + H/L)) prior to all analyses. General linear mixed models with wing length as a covariate (controlling for age/growth differences), weather indicators (T_mean_12_, T_max_12_, and T_min_12_ and P_12_) as independent variables, and year as a factor were run to test for differences in the H:L ratios between years (2015–2019); 2-way interactions were included and brood ID was used as a random effect. The above weather indicators we used covered 12 days of nestling life starting with the date of hatching in individual broods of pied flycatchers to test for an influence on the H:L ratio of nestlings during their period of high sensitivity. Degrees of freedom were approximated by the Satterthwaite method (Heck et al. 2010). The temperature indicators are closely related to each other, and therefore, they were tested in separate models in combination with precipitation. Thus, high or low mean ambient temperature is the result of high temperatures, including minimum and maximum temperatures, measured at equal intervals throughout the day. The initial model included the main independent variables, the year factor, and their two-way interaction terms. The model was simplified by step-wise deletion of non-significant interactions (Crawley 2002). Statistical analyses were performed using IBM SPSS 22 software (Heck et al. 2010). Coefficient estimates are presented with standard errors (SE). The results were considered significant at the level of 0.05.

## Results

**Fig. 1:**
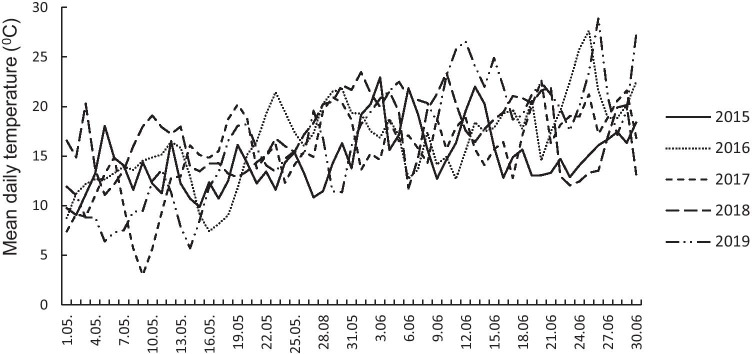
Mean daily temperature from 1.05 to 30.06 (seasons 2015–2019)

**Fig. 2:**
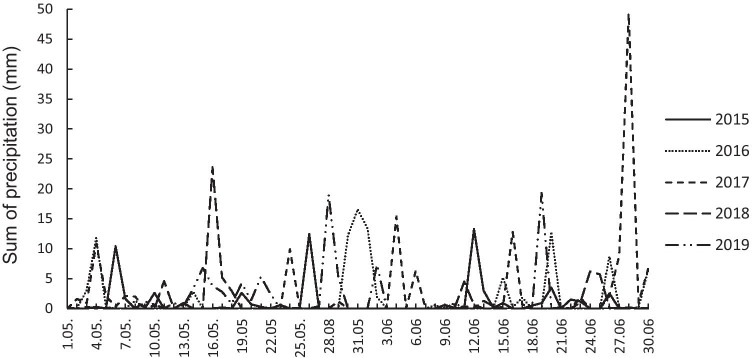
Total precipitation (mm) from 1.05 to 30.06 (seasons 2015–2019)

Both temperature and precipitation were highly variable during the study period, springs 2015–2019, especially during the early part of the flycatcher breeding season in the first half of May (Figs. 1 and 2). The coldest spring was in 2016 (12.94 °C), and the warmest in 2019 (16.98 °C); in 2018, the spring was driest (55.2 mm), whereas in 2017, it was most rainy (137.3 mm) (Figs. 1 and 2).

**Fig. 3:**
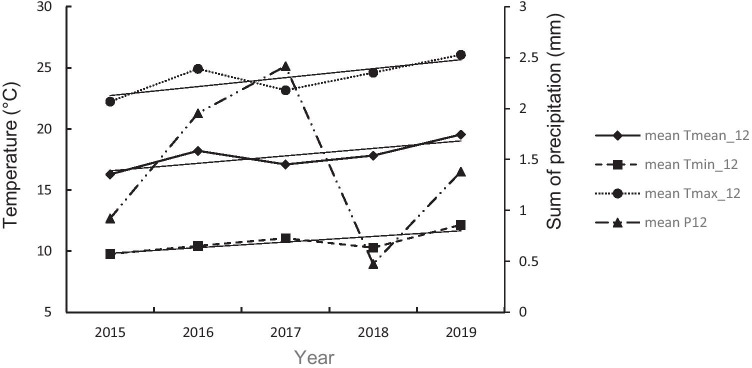
Annual mean values of individual pied flycatcher brood adjusted weather indicators for the first 12 days of nestling life: mean daily temperature (T_mean_12_), mean daily maximum temperature (T_max_12_), mean daily minimum temperature (T_min_12_), and total precipitation (P_12_) in 2015–2019, including temporal trend line for each temperature variable

We examined the influence of year, the temperature indicators (mean, the maximum, and minimum temperature for 12-day-nestling-period), the sum of precipitation, and wing length on the H:L ratio. Analyzing temperature differences (T_mean_12_, T_max_12_, T_min_12_) over 5 years (2015–2019), a similar pattern of variation between years was found for each temperature indicator (Fig. 3). Additionally, when analyzing each of the temperature indicators and the sum of precipitation for the first 12 days of the pied flycatcher nestling life, we found a statistically significant increase (an increasing trend) for each temperature (year × T_mean_12_ *r* = 0.57, *p* < 0.005; year × T_max_12_ *r* = 0.38, *p* = 0.002; year × T_min_12_ *r* = 0.45, *p* < 0.005), while the decrease for the sum of precipitation showed a decrease in 2015–2019 (year × P_12_ *r* =  − 0.44, *p* < 0.005) (Fig. 3). Temperature indicators were highest in 2019—T_mean_12_ = 19.05 °C; T_min_12_ = 12.20 °C; T_max_12_ = 26.10 °C and lowest in 2015—T_mean_12_ = 16.30 °C; T_min_12_ = 9.80 °C; T_max_12_ = 22.20 °C (Fig. 3). The between-year range of the minimum temperature indicator T_min_12_ was 2.40 °C, the range of daily mean temperature indicator T_mean_12_ was 3.25 °C, while the range of maximum temperature indicator T_max_12_ was 3.80 °C (Fig. 3). Total precipitation (P_12_) was highest in 2017 (2.42 mm) and lowest in 2018 (0.47 mm) (Fig. 3). Based on the data of each temperature indicator (T_mean_12_, T_max_12_, and T_min_12_), we found their constant slow increase during the 5 years of research. On the other hand, the sum of precipitation dropped as the temperatures increased (P_12_ × T_mean_12_ *r* =  − 0.43, *p* < 0.005; P_12_ × T_max_12_ *r* =  − 0.44, *p* < 0.005; P_12_ × T_min_12_ *r* =  − 0.26, *p* < 0.005).

All the temperature indicators covaried with the H:L ratio. Due to the fact that the T_min_12_ most strongly affected the H:L ratio, only this indicator is presented in detail (Table 1). In the final model, wing length and T_min_12_ and the 2-way interactions—year and wing length—were significant (Table 1), while neither year nor P_12_ had significant effects on the H:L-ratio. T_min_12_ had the highest effect on the increase in the H:L ratio in nestling’s blood (F_1,58.068_ = 5.253, *p* = 0.026, estimate ± SE = 0.013 ± 0.006; Table 1), while T_max_12_ caused the lowest increase in the stress index (F_1,58.186_ = 4.052, *p* = 0.049, estimate ± SE = 0.01 ± 0.005), with T_mean_12_ having an intermediate effect on increasing the H:L ratio (F_1, 58.790_ = 4.002, *p* = 0.045, estimate ± SE = 0.011 ± 0.006).

**Table 1:** General linear mixed models of H:L ratio of nestling pied flycatchers with wing length as a covariate, year as a factor and mean daily minimum temperature indicator (T_min_12_), and total precipitation indicator (P_12_) as independent variables; significant *p* values in the model are in bold. Deleted non-significant interactions are shown

		H/L ratio	
Effect	df	F	*P*
Final model			
Year	4. 56.994	1.560	0.197
Wing length (cov)	1. 95.093	9.504	**0.003**
T_min_12_ (cov)	1. 58.068	5.253	**0.026**
P_12_ (cov)	1. 56.902	0.220	0.641
Year * wing length	4. 92.070	2.534	**0.045**
Deleted terms			
T_min_12_ * P_12_	1. 51.662	0.005	0.942
Year * T_min_12_	4. 48.427	0.266	0.898
Wing length * _Tmin_12_	1. 110.331	0.130	0.719
Year * P_12_	4. 52.860	0.671	0.615
Wing length * P_12_	1. 100.013	1.426	0.235

**Fig. 4:**
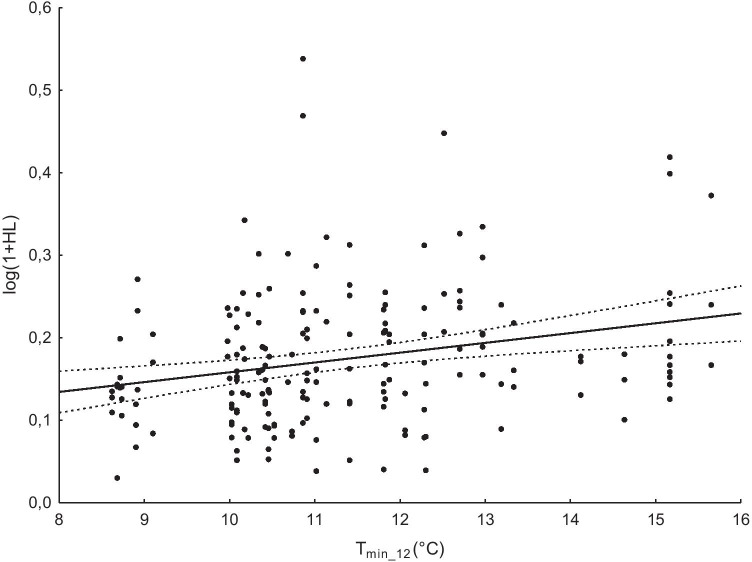
Relationship between the nestling H:L ratio and mean daily minimum temperature (T_min_12_) for individual broods of pied flycatchers

The wing length, as a co-covariate in interaction with temperature (T_mean_12_, T_max_12_, and T_min_12_), significantly affected the H:L ratio: wing length × T_min_12_ F_1,95.093_ = 9.504, *p* = 0.003, estimate ± SE =  − 0.005 ± 0.002 (Table 1); wing length × T_mean_12_ F_1,96.583_ = 9.606, *p* = 0.003, estimate ± SE =  − 0.006 ± 0.002; wing length × T_max_12_ F_1,97.191_ = 9.648, *p* = 0.002, estimate ± SE =  − 0.006 ± 0.002. In addition, the final model indicated that nestlings had a higher H:L ratio in response to increasing minimum temperature (T_min_12 1,58.068_ = 5.253, *p* = 0.026, estimate ± SE = 0.013425 ± 0.00586) (Fig. 4). There was a non-significant relationship between the level of the H:L ratio and P_12_ (P_12 1,56.902_ = 0.22, *p* = 0.641, estimates ± SE =  − 0.004 ± 0.0009; Table 1).

## Discussion

We found that temperature during the first 12 days of nestling development significantly positively affected the H:L ratio of nestling pied flycatchers, as shown by analyses of the temperature indicators: we found the strongest effects of T_min_12_ and weaker effects for T_mean_12_ and T_max_12_. This suggests that for pied flycatcher nestlings, an increase in the minimum ambient temperature caused a stress reaction reflected in an increase in the H:L ratio.

The increase in the H:L ratio results from nestlings’ sensitivity to increase ambient temperature as well as insufficient body thermoregulation (Rodríguez and Barba 2016). An increase in body temperature above normal and the dissipation of the heat produced by evaporation of water are the body’s main defense against thermal stress (Mertens 1977; O’Connor 1984). These mechanisms are very energy-consuming and can negatively affect body condition and lead to dehydration (Rodríguez and Barba 2016). An increase in the H:L ratio can also reflect a high level of corticosterone in the blood stream. Additionally, corticoids, including corticosterone, inhibit the function of the immune system, decrease serum protein concentrations, and increase the concentration of glucose in the blood (Bollengier-Lee et al. 1998). The large increase in ambient temperature may have a negative influence on the condition of nestlings (Belda et al. 1995; Sauve et al. 2021), but in our study, high temperatures appear to have relatively little effect on the H:L ratios, as they did not exceed 30 °C. In our climatic conditions, however, there may be large deviations from normal temperatures, which may be lower than average for the period. However, despite this, there is nevertheless an increasing trend in the ambient temperature including minimum temperature observed in our study area, which affects the stage of nestlings development. The effect of the increase in minimum temperatures on the H:L ratio may be due to the fact that the lower limit of thermal tolerance in pied flycatcher nestlings has not changed to adjust to the trend in ambient temperature, thus likely narrowing the optimal thermal range for nestling development. There is no very high increase in minimum temperatures in our study area, but even a small increase in minimum temperatures seemed sufficient for the temperatures to act as a stress factor for nestling flycatchers. Hence, even a small increase in minimum temperature significantly affected the health status of nestling pied flycatchers, which was manifested by an increase in the H:L ratio.

Heat stress resulting from environmental conditions may also lead to unrepairable thermoregulatory processes causing permanent muscle damage **(**Halevy et al. 2001**)** and even may be lethal to birds (Salaberria et al. 2014). Studies conducted on other hematological indicators also show a significant effect of temperature on these parameters in nestlings. Observations carried out on nestling great tits and blue tits show a significant effect of the minimum ambient temperature on the level of hemoglobin, an indicator of the nutritional condition of birds (Dunbar et al 2005), and glucose, an indicator allowing the assessment of the level of bird metabolism (Nadolski et al. 2006). The glucose concentration was negatively correlated with the minimum ambient temperature in great tit nestlings (Kaliński et al. 2014), while the hemoglobin level in young blue tits was positively correlated with minimum temperatures (Kaliński et al. 2015).

Although dehydration is a basic physiological process under elevated temperatures (> 25 °C), additional consequences may include changes in metabolic rate, oxidative stress, cardiovascular mortality, respiratory diseases, and thermoregulatory behavior (Shilov 1973; Salaberria et al. 2014; Rodríguez and Barba 2016). The increase in ambient temperature also contributes to a higher temperature inside nest-boxes especially in either sunny or too warm locations, causing nestling dehydration, low body mass, and delayed fledging (Imlay et al. 2019). This results in a lower chance of nestling survival in the nest as well as after fledging. This is confirmed by studies on nestling great tits, in which hyperthermia, at temperatures of 30–40 °C, caused a decrease in food intake (Greño et al. 2017), which slowed down the rate of muscle growth and development (Rodríguez and Barba 2016). Also, results on nestling great tits in south-eastern Spain show a negative relationship between minimum temperature and mean annual temperature and post-fledging survival probabilities (Greño et al. 2017). Similarly, a study on nestling tree swallows (*Tachycineta bicolor*) shows that the increase in nest temperature (> 35 °C) associated with an increase in ambient temperature during the first 12 days after hatching contributes to a decrease in reproductive success (Ardia 2013). The lower reproductive success of tree swallow nestlings at high temperatures may be due to a reduced ability to regulate body temperature, and temperatures above 35 °C impose a cost of their own to the body. However, in some cases, warm nest environments can reduce the cost of maintaining body temperature control (homeothermy) and thus promote faster growth (see Visser 1998; Rodríguez and Barba 2016). This has been confirmed in studies with tree swallow nestlings, where in experimentally heated nests young birds had faster body growth and a higher chance of survival to fledging (Dawson et al. 2005). Also, an experimental temperature increase in the nest to 31.8 °C allows energy to be used by nestlings to grow faster because less heat is discharged from the nest to the environment.

In our study on pied flycatcher nestlings, no effect of precipitation on the H:L ratio was observed. However, a number of experimental studies suggest that precipitation can significantly affect the leukocyte ratio in young birds. Thus, in young Eurasian kestrels (*Falco tinnunculus*), a significant effect of rainy periods on the H:L ratio was observed compared to periods without rain (Müller et al. 2011). Also in juvenile red kites (*Milvus milvus*), an increase in the H:L ratio was observed after heavy rainfall in the week before the study (Catitti 2018). From the observations by Catitti (2018), it can be concluded that precipitation may increase the sensitivity of young birds to infection by increasing the H:L ratio. And the increase of immune response, manifested by the increase of leukocyte ratio in the blood of nestlings, will help to counteract the development of infections and deterioration of condition. In addition, studies conducted on great tits show that during precipitation, adult birds decrease feeding, which is reflected in decreased body mass and condition in nestlings (Radford et al. 2001).

An imbalance between nestling development and unfavorable weather conditions and a reduction in the quality or quantity of food supplied during the breeding season may sometimes explain the differences in breeding success between seasons. Therefore, food availability and weather are the main factors influencing the growth rate and survival of nestlings (Catitti 2018). In our study on nestling pied flycatchers, a negative correlation between wing length and the H:L ratio was found, as well as a significant year-wing length interaction. In nestling barn swallows (*Hirundo rustica*), on the other hand, body weight and tarsus length were positively related to the ambient temperature on the day before the measurement was taken (Ambrosini et al. 2006). This is in line with the expectation that body growth is likely to be inhibited immediately with reduced food availability and that the metabolic costs of thermoregulation will increase (Cucco and Malacarne 1996a, b). Moreover, studies carried out on the serin (*Serinus serinus*) in the Mediterranean area show that high air temperatures during the breeding period can have a negative influence on the health and condition of nestlings (Belda et al. 1995). Warm and dry weather leads to a reduction in food resources (insects), which in turn reduces the health of the nestlings (Singer and Yom-Tov 1988).

Both thermal conditions and precipitation affect not only the reproductive success of birds but also the development and reproduction of ectoparasites (Merino and Potti 1995; Cumming and Van Vuuren 2006). Weather significantly affects nestling growth and survival in insectivorous bird populations (Merino and Potti 1996), and the interaction of poor weather conditions with nest-dwelling ectoparasites is particularly detrimental (Johnson and Albrecht 1993). However, observations by Marshall (1981) suggest that weather affects ectoparasite life more than it affects birds. Observations made on some ectoparasite species, such as the chicken mite (*Dermanyssus gallinae*), suggest that there is a significant effect of temperature on their development, growth, and activity (Marshall 1981; Maurer and Baumgärtner 1992). These observations are supported by a study conducted in nests of nestling blue tits, which involved increasing nest temperature by an average of 3 °C (Castaño-Vázquez et al. 2018). This experiment resulted in reduced numbers of mites and blowfly pupae (*Protocalliphora azurea*) in heated nests. In turn, an increase in minimum ambient temperature contributes to a decrease in the abundance of blackflies (Simuliidae), because they need running water to reproduce. An increase in ambient temperature causes small streams and watercourses to dry up (Martínez de la Puente et al. 2009). Also, precipitation has a significant effect on the activity and life processes (e.g., laying eggs, development of larvae) of ectoparasites. Studies conducted on fleas (*Ceratophyllus gallinae*) show that precipitation and low temperature contribute to a decrease in their activity (Bennett and Whitworth 1991; Rogers et al. 1991).

At temperate latitudes, spring and summer temperatures can vary considerably over several days, affecting the availability of insects and the metabolic costs of thermoregulation in small insectivorous birds (Turner 1982; Cucco and Malacarne 1996a, b). This has been confirmed by studies of McCarty and Winkler (1999) where ambient temperature influenced the abundance of aerial insects and thus indirectly body growth of nestling tree swallows. Unfavorable weather conditions (e.g., low temperatures, rainfall, or wind) are correlated with lower food availability or assimilability (Tulp and Schekkerman 2008) and the width of the food niche is reduced (Romanowski and Żmihorski 2008). A review of the literature reveals that nestlings of pied flycatchers receive food consisting of spiders (*Aranea*), beetles (*Coleoptera*), flies, and mosquitoes (*Diptera*), butterflies and moths (*Lepidoptera*), ants, bees, and wasps (*Hymenoptera*). Lepidoptera and Hymenoptera larvae are a major component of the diet of young pied flycatchers, while adult insects are more likely to be eaten from other insect groups (Lundberg and Alatalo 1992). Hence, this may be one of the reasons for an increase in the nestling H:L ratio with the progress of the breeding season in pied flycatchers. On the one hand, higher temperatures may cause an earlier breeding initiation time, which will be associated with fitting into the earlier peak of food biomass abundance. However, a higher nest temperature, associated with a higher ambient temperature, may contribute to faster growth of the body and thus increase the likelihood of young birds leaving the nest earlier and a greater chance of survival after fledging (Both et al. 1999).

Thermal conditions around the world are changing with global warming. This may significantly affect earlier phenology or bird migration. Faster phenology has altered the availability of insects during the period of peak food demand by nestlings. As a result of this mismatch, breeding at suboptimal food availability, birds suffer from poorer brood survival and lower nestling numbers. However, the flycatchers may attempt to achieve synchronization with food resources by initiating breeding earlier and reducing incubation duration (Visser et al. 1998). Furthermore, temperature not only affects reproductive efficiency and breeding success during the breeding season but also affects birds during the non-breeding season by influencing survival to the next reproductive period (Møller and Szép 2005; Szép et al. 2006), while long-distance migrants, such as pied flycatchers, arrive late to breeding grounds and migrate earlier as a result of climate change. In contrast, short-distance migratory birds may not only delay their return to the wintering grounds or stay on the breeding grounds, but they may also initiate breeding earlier and have an extended breeding season and good conditions on the breeding grounds (Schneider 2008). In contrast to short-distance migratory birds, long-distance migrants may not benefit from rising global temperatures. Their breeding period may not be extended, and breeding initiation is constrained by a fixed arrival date on the breeding grounds, which is controlled by endogenous rhythms (Both and Visser 2001). In contrast, the timing of return to the wintering grounds is not constrained by conditions on the breeding grounds but is determined by the onset of the dry season on the Sahel (Jenni and Kéry 2003). Also for the pied flycatcher, the ambient temperature along the migratory route north affects the time of return to the breeding grounds and the timing of the first egg laying (Both et al. 2006).

To sum up, an increase in ambient temperature had a significant effect on the condition of the nestling pied flycatchers, which was manifested by an increase in the H:L ratio as a response to heat stress. The increase in ambient temperature, including the minimum temperature, narrowed the optimum temperature range for nestlings and acted as a stressor. In addition, the increase in the H:L ratio resulted from the nestlings’ sensitivity, in the first 12 days of life, to the ambient temperature and was simultaneously an organismal response, e.g., stress and inadequate thermoregulation. Each of the analyzed temperature indicators caused an increase in the H:L ratio as a response of the organism to the stress factor, but the pied flycatcher nestlings were most affected by an increase in minimum temperature, and to a lesser extent by an increase in maximum temperature.

The collection of biological material from animals took place in accordance with Polish legal guidelines and was approved by the Local Ethical Committee for Animal Experiments in Lodz.
